# ﻿Complete mitochondrial genome of *Conuslischkeanus* Weinkauff, 1875 (Neogastropoda, Conidae) and phylogenetic implications of the evolutionary diversification of dietary types of *Conus* species

**DOI:** 10.3897/zookeys.1088.78990

**Published:** 2022-03-10

**Authors:** Yucheol Lee, Joong-Ki Park

**Affiliations:** 1 Marine Research Center, National Park Research Institute, Korea National Park Service, 1 Bakramhow-gil, Yeosu-si, Jeollanam-do, Republic of Korea National Park Research Institute, Korea National Park Service Yeosu-si Republic of Korea; 2 Division of EcoScience, Ewha Womans University, 52 Ewhayeodae-gil, Seodaemun-gu, Seoul, Republic of Korea Ewha Womans University Seoul Republic of Korea

**Keywords:** Cone snail, dietary type evolution, Lividoconus

## Abstract

The family Conidae, commonly known as cone snails, is one of the most intriguing gastropod groups owing to their diverse array of feeding behaviors (diets) and toxin peptides (conotoxins). *Conuslischkeanus* Weinkauff, 1875 is a worm-hunting species widely distributed from Africa to the Northwest Pacific. In this study, we report the mitochondrial genome sequence of *C.lischkeanus* and inferred its phylogenetic relationship with other *Conus* species. Its mitochondrial genome is a circular DNA molecule (16,120 bp in size) composed of 37 genes: 13 protein-coding genes (PCGs), 22 transfer RNA genes, and two ribosomal RNA genes. Phylogenetic analyses of concatenated nucleotide sequences of 13 PCGs and two ribosomal RNA genes showed that *C.lischkeanus* belongs to the subgenus Lividoconus group, which is grouped with species of the subgenus Virgiconus, and a member of the largest assemblage of worm-hunting (vermivorous) species at the most basal position in this group. Mitochondrial genome phylogeny supports the previous hypothesis that the ancestral diet of cone snails was worm-hunting, and that other dietary types (molluscivous or piscivorous) have secondarily evolved multiple times from different origins. This new, complete mitochondrial genome information provides valuable insights into the mitochondrial genome diversity and molecular phylogeny of *Conus* species.

## ﻿Introduction

The genus *Conus* Linnaeus, 1758 is a well-known predatory gastropod group that produces venomous peptides, called conotoxins, to capture prey and defend against predators (Dutertre et al. 2004; Prashanth et al. 2016; Kohn 2019). There are more than 750 *Conus* species reported worldwide (WoRMS 2021), which are widely distributed in tropical and subtropical ocean areas in various environments ranging from deep water to the intertidal zone (Kohn 1959). With the notable exception of a few conid species that prey on more than one dietary type (e.g., *Californiconuscalifornicus* (Reeve, 1844) and *Conusbullatus* Linnaeus, 1758), most species in this genus show a very narrow range of prey, feeding on worms, mollusks, and fishes, and they are grouped into three specialized dietary types according to their prey types: vermivorous (worm-hunting), molluscivorous (mollusk-hunting), and piscivorous (fish-hunting) (Duda Jr et al. 2001; Olivera et al. 2014; Robinson et al. 2014; Himaya et al. 2015; Gao et al. 2018). Among these diverse dietary types, the worm-hunting diet is the most common, accounting for more than 70% of the species, and it is widely considered the most ancestral; other dietary types are regarded to have undergone secondary evolution (Duda Jr et al. 2001; Puillandre et al. 2014; Gao et al. 2018; Abalde et al. 2019). The evolutionary origin and diversification of their dietary specification can be better understood based on well-reconstructed phylogenetic relationships among *Conus* species of different diet types.

The implementation of new sequencing technologies (e.g., next-generation sequencing; NGS) and various bioinformatics tools has allowed mitochondrial genome sequencing to be markedly easier, cost-effective, and widely used for studying phylogeny in various metazoan groups, including *Conus* species (Abalde et al. 2017; Uribe et al. 2017, 2018). As of January 2022, complete and partial mitochondrial genome sequences of 60 *Conus* species have been reported in GenBank, most of which are tropical and subtropical species, and diverse species in other oceanic regions are relatively underrepresented. To elucidate the phylogenetic relationships and evolution of dietary specialization within the genus, phylogenetic analysis using additional mitochondrial genome information sampled from various regional species is needed. To date, only partial mitochondrial gene sequences (12S, 16S, and *cox1*) of *Conuslischkeanus* are currently available on GenBank, with no complete mitochondrial genome information for this species. *Conuslischkeanus* Weinkauff, 1875 is a vermivorous species reported from East Africa to the western Pacific (Röckel et al. 1995). In this study, we determine the complete mitochondrial genome of *C.lischkeanus* for the first time and perform a phylogenetic analysis of 13 protein-coding genes and two ribosomal RNA (rRNA) gene sequences of 39 *Conus* species with different dietary types, including *C.lischkeanus*.

## ﻿Materials and methods

### ﻿Sample collection and DNA extraction

*Conuslischkeanus* specimen was collected from Moonseom, Jeju Island, Korea, preserved in 95% ethanol solution, and deposited in the Marine Mollusk Resource Bank of Korea (MMRBK; voucher specimen no. MMRBK6746) in Seoul, Korea. The specimen was morphologically identified based on shell characters, which include a conical last whorl covered with yellow-brown periostracum and an angular shoulder. Total genomic DNA was extracted from the foot tissue using an E.Z.N.A. mollusc DNA kit (Omega Bio-tek, Norcross, GA, USA) following the manufacturer’s instructions.

### ﻿NGS and mitochondrial genome assembly and annotation

Whole-genome sequencing libraries were prepared using the MGIEasy DNA library prep kit (BGI, Shenzhen, China) according to the manufacturer’s instructions and quantified using the QuantiFluor ssDNA System (Promega Corporation, Madison, WI, USA). Sequencing was conducted on the MGISEQ-2000 system with 150 base-pair reads. A total of 48,608,637 raw reads were obtained, and adapter-trimmed using a skewer program (Jiang et al. 2014) with a mean quality threshold of 20. The mitochondrial genome was assembled from trimmed reads using MITObim v. 1.9.1 (Hahn et al. 2013). Mitochondrial gene annotation was performed using MITOS websever (Bernt et al. 2013) and confirmed through sequence comparison with mitochondrial genomes of other *Conus* species previously reported (Bandyopadhyay et al. 2008; Cunha et al. 2009; Brauer et al. 2012; Barghi et al. 2016; Chen et al. 2016a, 2016b, 2016c; Gao et al. 2018; Uribe et al. 2018) using Geneious v. 9.1.8 (Kearse et al. 2012). The nucleotide composition, amino acid composition, and relative synonymous codon usage (RSCU) were analyzed using the MEGA X program (Kumar et al. 2018). Nucleotide composition skew was calculated using the following formula: AT-skew = [A – T] / [A + T] and GC-skew = [G – C] / [G + C] (Perna and Kocher 1995).

### ﻿Phylogenetic analysis

To determine the relationship between *C.lischkeanus* and other *Conus* species, phylogenetic analyses were performed for the nucleotide sequences of 13 protein-coding genes (PCGs) and two rRNA genes from 39 complete or nearly complete mitochondrial genomes of the family Conidae (Table 1). *Tomopleura* sp., belonging to the family Borsoniidae, was also included as an outgroup in the analysis. A concatenated nucleotide sequence dataset (13,870 bp long) of the 13 PCGs and two rRNA genes was prepared for phylogenetic analysis. The best substitution model for each gene was estimated using jModelTest v. 2.1.10 (Darriba et al. 2012) with the Akaike information criterion (AIC) for the nucleotide dataset. Phylogenetic analyses were conducted using maximum likelihood (ML) and Bayesian inference (BI) methods. ML analysis was performed using RAxML v. 8.2.9 (Stamatakis 2014) with a heuristic search and 10,000 bootstrap replicates. The BI tree was generated using the Markov chain Monte Carlo method, with two independent runs of 1 × 10^6^ generations with four chains, sampling every 100 generations and discarding the first 25% generations as burn-in. Both ML and BI programs were conducted using the CIPRES portal (Miller et al. 2010).

**Table 1.** Complete mitochondrial genomes used for phylogenetic analysis in this study.

Family	Species	Diet	GenBank	Reference
Conidae	*Conusvictoriae*	Molluscivorous	—	Abalde et al. 2019
	*Conusgloriamaris*	Molluscivorous	KU996360	—
	*Conustextile*	Molluscivorous	DQ862058	Bandyopadhyay et al. 2008
	*Conusepiscopatus*	Molluscivorous	—	Abalde et al. 2019
	*Conusmarmoreus*	Molluscivorous	—	Abalde et al. 2019
	*Conusnobilis*	Molluscivorous	KX263253	Uribe et al. 2017
	*Conusermineus*	Piscivorous	KY864977	Abalde et al. 2017
	*Conustulipa*	Piscivorous	KR006970	Chen et al. 2016a
	*Conusconsors*	Piscivorous	KF887950	Brauer et al. 2012
	*Conusstriatus*	Piscivorous	KX156937	Chen et al. 2016b
	*Conusbetulinus*	Vermivorous	—	Abalde et al. 2019
	*Conussponsalis*	Vermivorous	—	Abalde et al. 2019
	*Conusarenatus*	Vermivorous	—	Abalde et al. 2019
	*Conusgoudeyi*	Vermivorous	KY864975	Abalde et al. 2019
	*Conusebraeus*	Vermivorous	—	Abalde et al. 2019
	*Conuscoronatus*	Vermivorous	—	Abalde et al. 2019
	*Conusmiliaris*	Vermivorous	—	Abalde et al. 2019
	*Conuspseudonivifer*	Vermivorous	KY864969	Abalde et al. 2017
	*Conusvenulatus*	Vermivorous	KX263250	Uribe et al. 2017
	*Conusateralbus*	Vermivorous	KY864970	Abalde et al. 2017
	*Conusbyssinus*	Vermivorous	KY864973	Abalde et al. 2017
	*Conuspulcher*	Vermivorous	KY864972	Abalde et al. 2017
	*Conusgenuanus*	Vermivorous	KY864974	Abalde et al. 2019
	*Conushybridus*	Vermivorous	KX263252	Uribe et al. 2017
	*Conusguanche*	Vermivorous	KY801847	Abalde et al. 2017
	*Conusventricosus*	Vermivorous	KX263251	Uribe et al. 2017
	*Conusmiruchae*	Vermivorous	KY864971	Abalde et al. 2017
	*Conusborgesi*	Vermivorous	EU827198	Cunha et al. 2009
	*Conusinfinitus*	Vermivorous	KY864967	Abalde et al. 2017
	*Conusspurius*	Vermivorous	KY864976	Abalde et al. 2019
	*Conusvirgo*	Vermivorous	—	Abalde et al. 2019
	*Conusquercinus*	Vermivorous	KY609509	Gao et al. 2018
	*Conuslischkeanus*	Vermivorous	OL632021	This study
	*Conuslividus*	Vermivorous	—	Abalde et al. 2019
	*Conustabidus*	Vermivorous	KY864968	Abalde et al. 2019
	*Conuslenavati*	Vermivorous	—	Abalde et al. 2019
	*Conustribblei*	Vermivorous	KT199301	Barghi et al. 2016
	*Conusimperialis*	Vermivorous	—	Abalde et al. 2019
	*Conuscapitaneus*	Vermivorous	KX155573	Chen et al. 2016c
	*Conasprellawakayamaensis*	Vermivorous	KX263254	Uribe et al. 2017
	*Californiconuscalifornicus*	All	KX263249	Uribe et al. 2017
	*Profundiconusteramachii*	Vermivorous	KX263256	Uribe et al. 2017
Borsoniidae	*Tomopleura* sp.	—	KX263259	Uribe et al. 2017

## ﻿Results and discussion

### ﻿Mitochondrial genome organization and nucleotide composition

**Figure 1.**
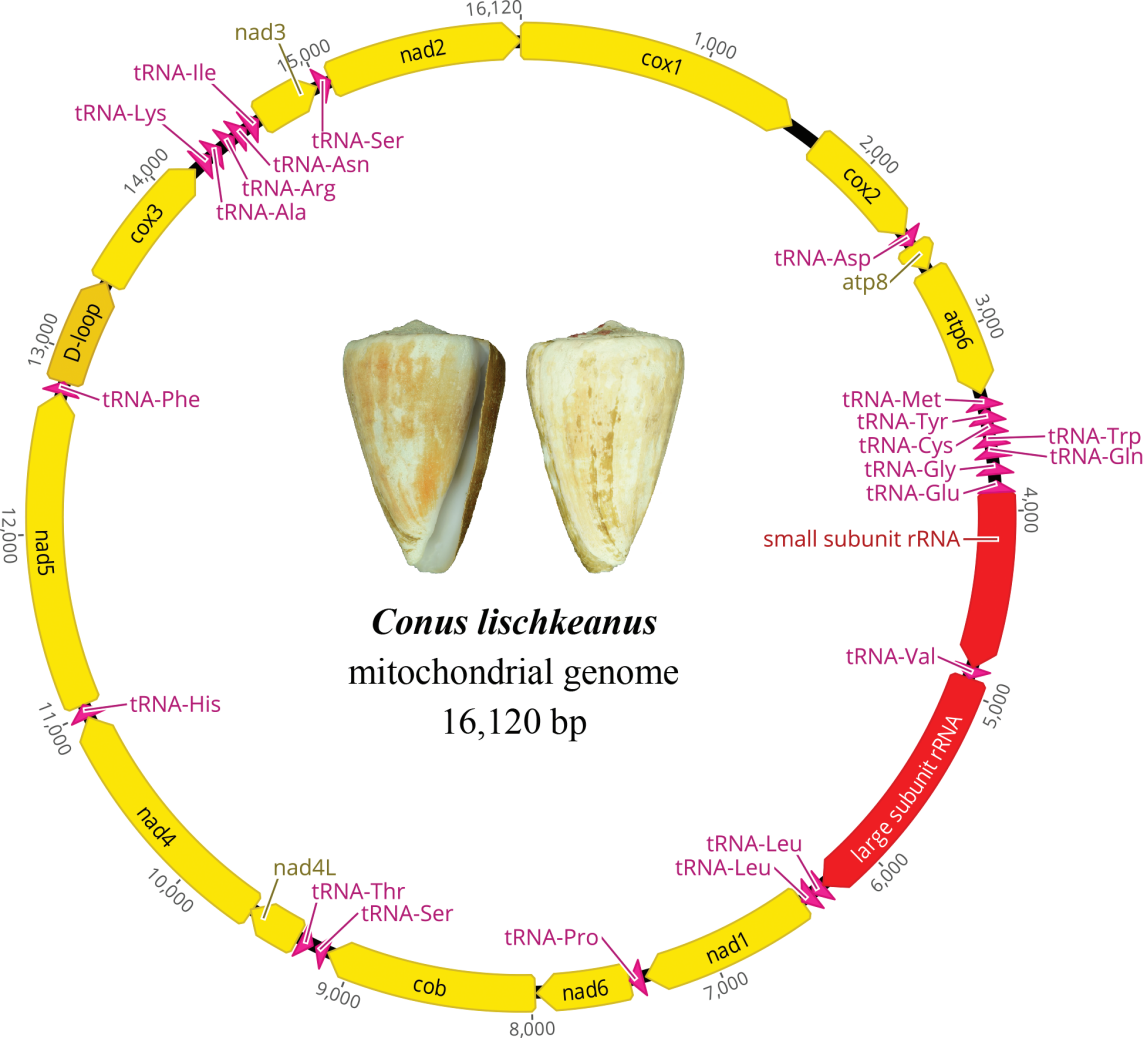
Mitochondrial genome structure of *Conuslischkeanus*.

*Conuslischkeanus* is widely distributed from East Africa to the western Pacific (Röckel et al. 1995), extending to Taiwan, Japan, and Korea (Jeju Island). This species shows a wide range of shell morph and color variations, depending on geographic origin, which were previously classified as a few separate subspecies (Coomans and Filmer 1985) but are now treated as local variations of *C.lischkeanus* (Röckel et al. 1995). In this study, we determine the complete mitochondrial genome of *C.lischkeanus* and compare it with other cone snail species to infer the evolutionary diversification of different dietary types. The complete mitochondrial genome of *C.lischkeanus* (GenBank accession number: OL632021) is 16,120 bp in size, encoding 13 PCGs, 22 tRNA genes, two rRNA genes, and one control region (Fig. 1, Table 2). The overall nucleotide base composition is 29% A, 37.1% T, 17.6% G, and 16.3% C (Table 3). All 13 PCGs, 14 tRNAs, and two rRNA genes are encoded on the heavy strand, whereas eight tRNA genes (*trnT*, *trnM*, *trnY*, *trnC*, *trnW*, *trnQ*, *trnG*, and *trnE*) are encoded on the light strand. The gene order is identical to that of other cone snail species, suggesting that the mitochondrial gene order of this genus is highly conserved (Bandyopadhyay et al. 2008; Cunha et al. 2009; Brauer et al. 2012; Barghi et al. 2016; Chen et al. 2016a, 2016b, 2016c; Gao et al. 2018; Uribe et al. 2018). The AT and GC-skew values of the entire genome sequences, which represent the measures of compositional asymmetry, were negative (−0.1233) and positive (0.0390), respectively, similar to those of cone snails (Gao et al. 2018).

**Table 2.** Gene regions in the mitochondrial genome of *Conuslischkeanus*.

Gene	Start	Stop	Strand direction	Length (bp)	Codon (start)	Codon (stop)	Overlapping regions	Intergenic spacers
*cox1*	1	1,548	H	1,548	ATG	TAA	1	166
*cox2*	1,715	2,401	H	687	ATG	TAA	—	—
tRNA-Asp (*trnD*) (gtc)	2,402	2,468	H	67			—	—
*atp8*	2,469	2,630	H	162	ATG	TAA	—	6
*atp6*	2,637	3,359	H	723	ATG	TAA	—	11
tRNA-Met (*trnM*) (cat)	3,371	3,438	L	68			—	12
tRNA-Tyr (*trnY*) (gta)	3,451	3,516	L	66			—	1
tRNA-Cys (*trnC*) (gca)	3,518	3,582	L	65			—	—
tRNA-Trp (*trnW*) (tca)	3,583	3,648	L	66			—	—
tRNA-Gln (*trnQ*) (ttg)	3,646	3,711	L	66			3	24
tRNA-Gly (*trnG*) (tcc)	3,736	3,802	L	67			—	35
tRNA-Glu (*trnE*) (ttc)	3,838	3,902	L	65			—	—
small subunit rRNA (*rrnS*)	3,903	4,854	H	952			—	—
tRNA-Val (*trnV*) (tac)	4,855	4,921	H	67			—	—
large subunit rRNA (*rrnL*)	4,922	6,296	H	1,375			—	—
tRNA-Leu1 (*trnL1*) (tag)	6,297	6,366	H	70			—	6
tRNA-Leu2 (*trnL2*) (taa)	6,373	6,441	H	69			—	—
*nad1*	6,442	7,383	H	942	ATG	TAG	—	16
tRNA-Pro (*trnP*) (tgg)	7,400	7,468	H	69			—	—
*nad6*	7,469	7,975	H	507	ATG	TAA	—	13
*cob*	7,989	9,128	H	1,140	ATG	TAA	—	11
tRNA-Ser2 (*trnS2*) (tga)	9,140	9,204	H	65			—	16
tRNA-Thr (trnT) (tgt)	9,221	9,289	L	69			—	22
*nad4L*	9,312	9,608	H	297	ATG	TAG	—	—
*nad4*	9,602	10,984	H	1,383	ATG	TAG	7	—
tRNA-His (*trnH*) (gtg)	10,984	11,049	H	66			1	—
*nad5*	11,050	12,765	H	1,716	ATG	TAA	—	—
tRNA-Phe (*trnF*) (gaa)	12,765	12,829	H	65			1	—
D-loop	12,830	13,415	H	586			—	—
*cox3*	13,416	14,195	H	780	ATG	TAA	—	34
tRNA-Lys (*trnK*) (ttt)	14,230	14,298	H	69			—	9
tRNA-Ala (*trnA*) (tgc)	14,308	14,374	H	67			—	22
tRNA-Arg (*trnR*) (tcg)	14,397	14,465	H	69			—	11
tRNA-Asn (*trnN*) (gtt)	14,477	14,545	H	69			—	12
tRNA-Ile (*trnI*) (gat)	14,558	14,626	H	69			—	5
*nad3*	14,632	14,985	H	354	ATG	TAA	—	15
tRNA-Ser1 (*trnS1*) (gct)	15,001	15,068	H	68			—	—
*nad2*	15,069	1	H	1,053	ATG	TAA	—	—

### ﻿PCGs and codon usage

**Figure 2.**
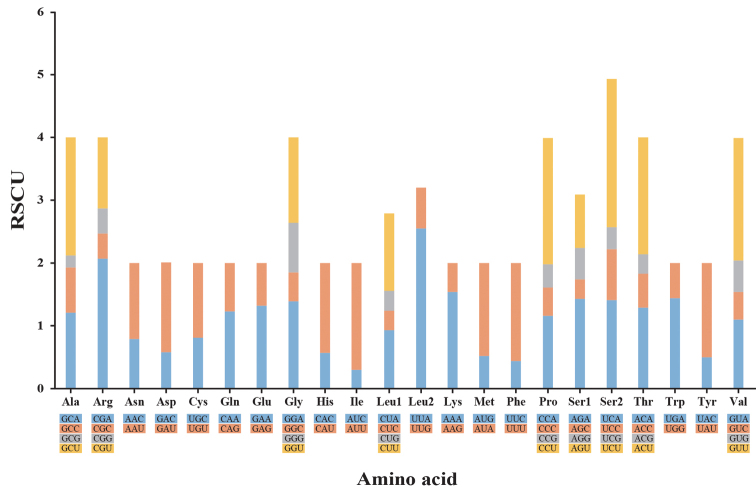
The relative synonymous codon usage (RSCU) frequency of the mitochondrial genome of *Conuslischkeanus*.

The lengths of 13 PCGs of *C.lischkeanus* mitochondria range from 162 bp (*atp8*) to 1,716 bp (*nad5*) and contain 3,751 codons, excluding termination codons. The base composition of PCGs is 26.3% A, 39.2% T, 17.5% G, and 17.0% C, and the overall AT content was 65.5%, which is very similar to that of the entire mitochondrial genome sequence (AT content of 66.1%; Table 3). All PCGs have ATG as the initiation codon. With the exception of three PCGs (*nad1*, *nad4L*, and *nad4*) with TAG as a termination codon, all PCGs have TAA as a termination codon, which is consistent with complete mitochondrial genomes previously reported (Bandyopadhyay et al. 2008; Cunha et al. 2009; Brauer et al. 2012; Barghi et al. 2016; Chen et al. 2016a, 2016b, 2016c; Gao et al. 2018; Uribe et al. 2018). Fig. 2 shows the RSCU of *C.lischkeanus*, wherein the five most frequently used codons are UUA (Leu1), UCU (Ser2), CGA (Arg), CCU (Pro), and GUU (Val). In addition, codons with an A or U in the third position are the most frequently used, which is consistent with observations made in other mollusk species (Rawlings et al. 2010; Ren et al. 2010; Lee et al. 2019).

**Table 3.** Nucleotide composition of the mitochondrial genome of *Conuslischkeanus*.

Nucleotide sequence	Length (bp)	A (%)	C (%)	G (%)	T (%)	A+T (%)	G+C (%)
Entire sequence	16,120	29.0	16.3	17.6	37.1	66.1	33.9
Protein coding sequence	11,292	26.3	17.0	17.5	39.2	65.5	34.5
Codon position*							
1^st^	3,751	26.9	17.2	24.7	31.2	58.1	41.9
2^nd^	3,751	18.3	20.9	16.6	44.2	62.5	37.5
3^rd^	3,751	33.4	13.1	11.4	42.1	75.5	24.5
Ribosomal RNA gene sequence	2,327	35.5	14.4	18.4	31.6	67.2	32.8
Transfer RNA gene sequence	1,481	34.0	16.2	17.7	32.1	66.1	33.9
D-loop region sequence	586	31.1	15.4	17.6	35.8	67.1	32.9

*Termination codons were not included.

### ﻿tRNA, rRNA genes, and D-loop regions

**Figure 3.**
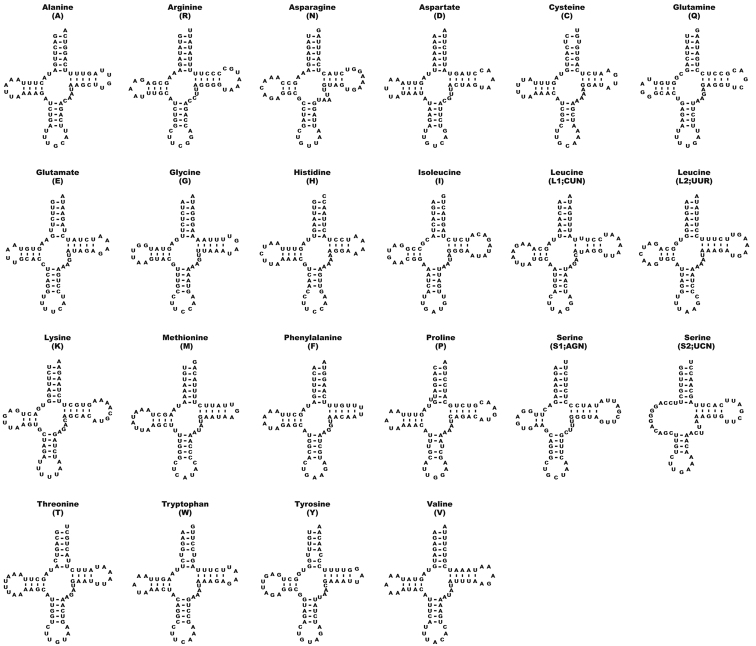
Predicted tRNA structures of *Conuslischkeanus*.

Twenty-two tRNA genes were found in the mitochondrial genome of *C.lischkeanus*. The length of tRNA genes range from 65 bp (*trnC*, *trnE*, *trnS2*, and *trnF*) to 70 bp (*trnL1*) (Table 2). All tRNA genes formed typical clover-leaf secondary structures, except for *trnS1* and *trnS2* which lack or had an imperfect D-arm (Fig. 3), which is common to other mollusk species (Boore 2006; Feng et al. 2020). Meanwhile, two ribosomal RNA genes with a total length of 2,327 bp consisting of small rRNA (*rrnS*; 952 bp) and large rRNA (*rrnL*; 1,375 bp) are located between *trnE* and *trnV*, and between *trnV* and *trnL1*, respectively (Fig. 2, Table 2). The D-loop is 587 bp in length and is located between *trnF* and *cox3*, with a short, inverted repeat (IR1; 20 bp), a typical feature of the mitochondrial genome of cone snail species. In contrast, the AT tandem repeat stretch found in *C.consors* G. B. Sowerby I, 1833 and *C.quercinus* [Lightfoot], 1786 was not identified in the *C.lischkeanus* mitochondrial genome (Brauer et al. 2012; Gao et al. 2018).

### ﻿Phylogenetic implication of the evolutionary diversification of dietary specification

**Figure 4.**
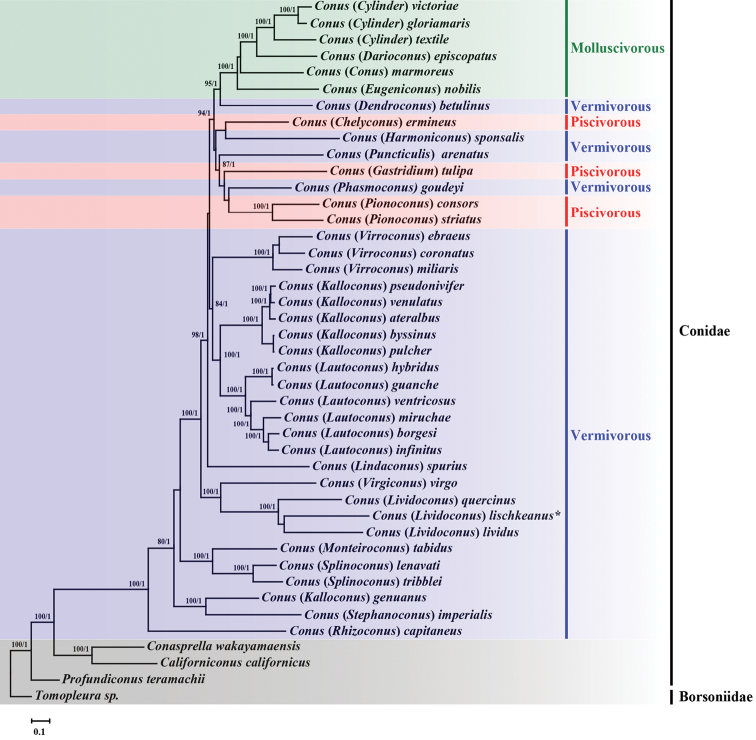
Phylogenetic relationships of the genus *Conus* based on concatenated nucleotide sequences (13 protein coding genes plus two rRNA genes). Numbers above branches are statistical support values for ML (bootstrap values, > 70)/BI (posterior probability values, > 0.7). *: determined in this study.

Phylogenetic analysis using ML and BI methods yield similar results with respect to the tree topology, as shown in Fig. 4. All subgenera, except *Kalloconus* da Motta, 1991, were monophyletic. A group of three *Conus* species, (*C.capitaneus*+(*C.imperialis*+*C.genuanus*)) was positioned at the most basal, but the branch reflected relatively weak supporting values (< 70% bootstrap values). Instead, the next monophyletic group consisting of (*C.tabidus*+(*C.lenavati*+*C.tribblei*)) was strongly supported (100% in ML and 1.0 BPP). Moreover, three species belonging to the subgenus Lividoconus Wils, 1970 (including *C.lischkeanus*) were grouped together with the subgenus Virgiconus Cotton, 1945 species *C.virgo* Linnaeus, 1758, a sister to a large assemblage of the remaining *Conus* species that is composed of two well-supported groupings differing in their feeding type: vermivorous species and a mixture of three diet types. The “vermivorous only” clade is composed of three monophyletic groups of the subgenera *Virroconus* Iredale, 1930, *Kalloconus* da Motta, 1991, and *Lautoconus* Monterosato, 1923, with the latter two more closely related to each other than to *Virroconus*. Within the “mixed diet” clade, aside from a well-supported molluscivorous species (100% BP in ML and 1.0 BPP in BI), all vermivorous species are grouped either with piscivorous or vermivorous species. It is evident that vermivorous species are not monophyletic and are split into four branches, each forming sister relationships with other molluscivorous and/or piscivorous species. Given the mitochondrial genome phylogeny with vermivorous species positioned at the basal, the tree topology coincides with earlier hypothesis that worm-hunting was the ancestral diet type. Meanwhile, the other two diet types such as molluscivorous and piscivorous were secondarily derived (Duda Jr et al. 2001; Puillandre et al. 2014; Gao et al. 2018; Abalde et al. 2019). Notably, piscivorous species in our phylogenetic tree are not monophyletic and split into three branches, which is not consistent with previous mitochondrial genome phylogeny where fish-hunting species formed a monophyletic group (Gao et al. 2018). The polyphyly of piscivorous species in the current study implies that the fish-hunting species have evolved independently from worm-hunting groups multiple times. The complete mitochondrial genome information of the worm-hunting *Conus* species (*C.lischkeanus*) in the present study provides valuable insights into the mitochondrial genome diversity and molecular phylogeny of *Conus* species.
